# Association between destination hospital level and outcomes in out-of-hospital cardiac arrest patients under a dispatch-guided transport system: a population-based study in Nagoya, Japan

**DOI:** 10.1016/j.resplu.2026.101482

**Published:** 2026-09-08

**Authors:** Akihiro Kato, Asako Matsushima, Momoko Sugiyama, Akito Kubota, Kazunori Imai, Toshiyasu Miura, Yasumasa Ode

**Affiliations:** Department of Emergency and Critical Care, Nagoya City University Graduate School of Medical Sciences, 1-2-23 Wakamizu, Chikusa-ku, Nagoya, Aichi 464-8547, Japan

**Keywords:** Out-of-hospital cardiac arrest, Destination hospital, Critical care medical center, Dispatch-guided transport, Refractory shockable rhythm

## Abstract

**Background:**

The relationship between destination hospital level and outcomes in out-of-hospital cardiac arrest (OHCA) patients remains controversial. To examine the association between transport to critical care medical centers and outcomes, we analyzed all city-wide data from Nagoya City, where the initial hospital request is made without consideration of initial cardiac rhythm.

**Methods:**

This retrospective cohort study included 12,663 OHCA patients in Nagoya City between April 2019 and March 2025. Patients were classified by destination hospital: critical care medical center (*n* = 11,149) or other hospital (*n* = 1514). Favorable one-month neurological outcomes and one-month survival were evaluated. Inverse probability of treatment weighting (IPTW) evaluated associations between destination hospital level and outcomes. Refractory shockable rhythm was explored.

**Results:**

In the critical care medical center and other hospital groups, favorable one-month neurological outcome rates were 1.9% versus 1.7% (*P* = 0.683), and survival rates were 4.1% versus 4.5% (*P* = 0.494). IPTW showed no association between destination hospital level and favorable neurological outcomes overall or by rhythm. Transport to critical care medical centers was associated with lower one-month survival in patients with non-shockable rhythm (odds ratio, 0.66; 95% confidence interval, 0.46–0.95). Exploratory analysis showed higher one-month survival among patients with refractory shockable rhythm transported to critical care medical centers (19.7% vs. 3.3%), although the estimate was imprecise.

**Conclusion:**

Under a dispatch-guided transport system, transport to critical care medical centers was not associated with favorable neurological outcomes in either rhythm group. These findings do not establish a preferred transport strategy, although selected patients may have better outcomes at these centers.

## Highlights

• After OHCA calls, EMS dispatch rapidly requested the nearest accepting hospital.
• Initial request did not consider hospital level, rhythm, or bystander intervention.
• Critical care medical center transport was not associated with better outcomes.
• Higher survival was observed in refractory shockable OHCA at critical care centers.

## Graphical abstract

**Figure:**
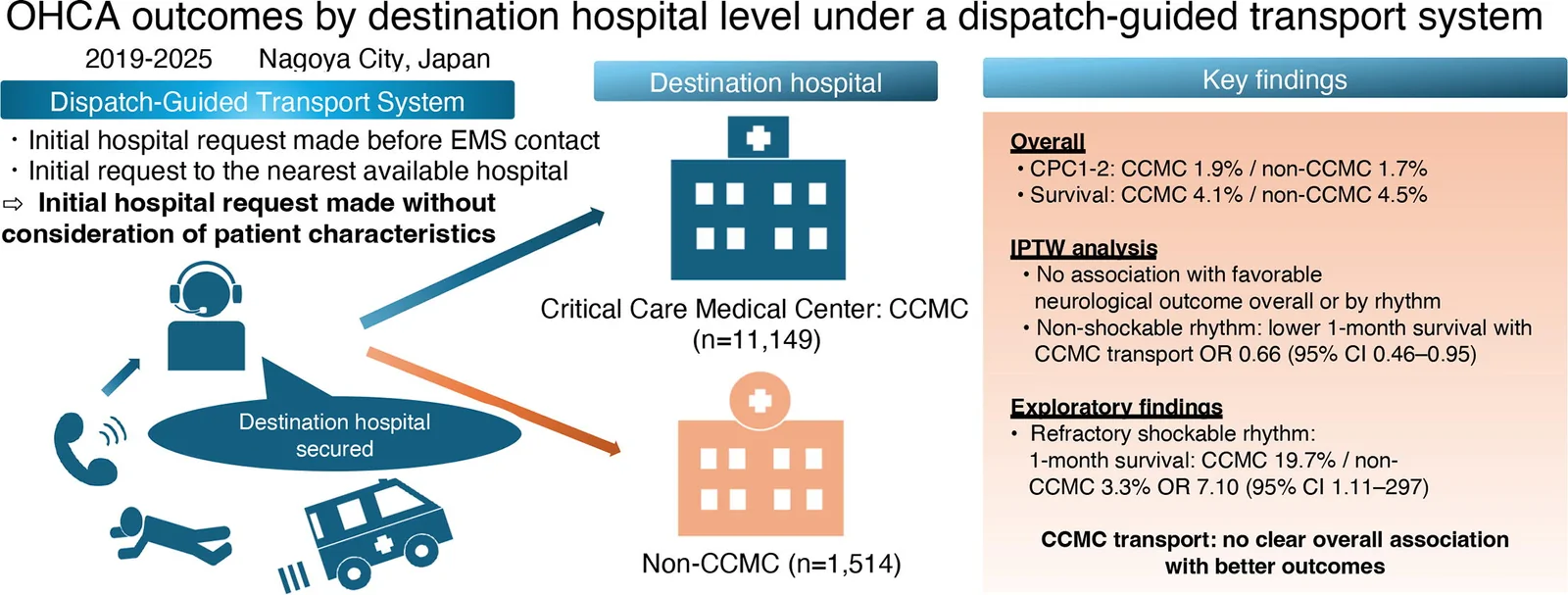

## Introduction

As populations age, the incidence of out-of-hospital cardiac arrest (OHCA) is increasing worldwide.^1^, ^2^, ^3^ However, outcomes remain poor, with reported one-month and one-year survival rates of 10.7% and 7.7%, respectively, among adult patients with OHCA.^4^

Recent guidelines recommend the transport of patients with OHCA to cardiac arrest centers capable of providing percutaneous coronary intervention (PCI) and comprehensive post-resuscitation care.^5^ However, centralization of OHCA care may also prolong transport time and concentrate the burden on higher-level hospitals, and it remains unclear whether uniform transport of all patients with OHCA to higher-level centers is optimal for either patients or regional emergency care systems.^6^, ^7^ Previous registry-based studies have suggested that younger patients and those with shockable rhythms are more likely to be transported to higher-level hospitals and to have better outcomes.^8^, ^9^ However, these results were based on registry studies, and the generalizability of their findings to the whole population remains controversial.

Nagoya City, located in central Japan, is a metropolitan area with a population of 2.33 million and an area of approximately 326.5 km^2^, where approximately 2300 cases of OHCA occur annually.^10^ To facilitate rapid transport of critically ill patients, including those with OHCA or shock, a dispatch-guided destination decision system has been adopted. Under this system, the dispatch center requests the nearest hospital to accept OHCA patients before the emergency medical team arrives at the scene. The initial request is made regardless of patient characteristics or initial electrocardiographic rhythm.

In this study, we aimed to examine the association between destination hospital level and one-month neurological outcomes and survival among all OHCA patients transported under this dispatch-guided system in Nagoya City.

## Methods

### Study design and setting

This was a retrospective population-based cohort study of patients with OHCA in Nagoya City, Japan, using Utstein-style data recorded by the Nagoya City Fire Bureau. This study was approved by the Institutional Review Board of Nagoya City University on January 5, 2026 (approval reference number: 60-25-0160). Because anonymized data were used, the requirement for informed consent was waived and an opt-out approach was adopted. Opt-out information was posted on the institutional website, allowing data removal before anonymization.

### Study population

We identified 14,115 patients with OHCA who were transported by emergency medical services (EMS) in Nagoya City between April 1, 2019, and March 31, 2025. Of these, 1452 patients who developed cardiac arrest after the emergency call were excluded, leaving 12,663 patients for the final analysis (Fig. 1). Patients who progressed to cardiac arrest after the emergency call were excluded because the underlying pathophysiology and timing of EMS intervention differed from those of other OHCA cases.

**Fig. 1:**
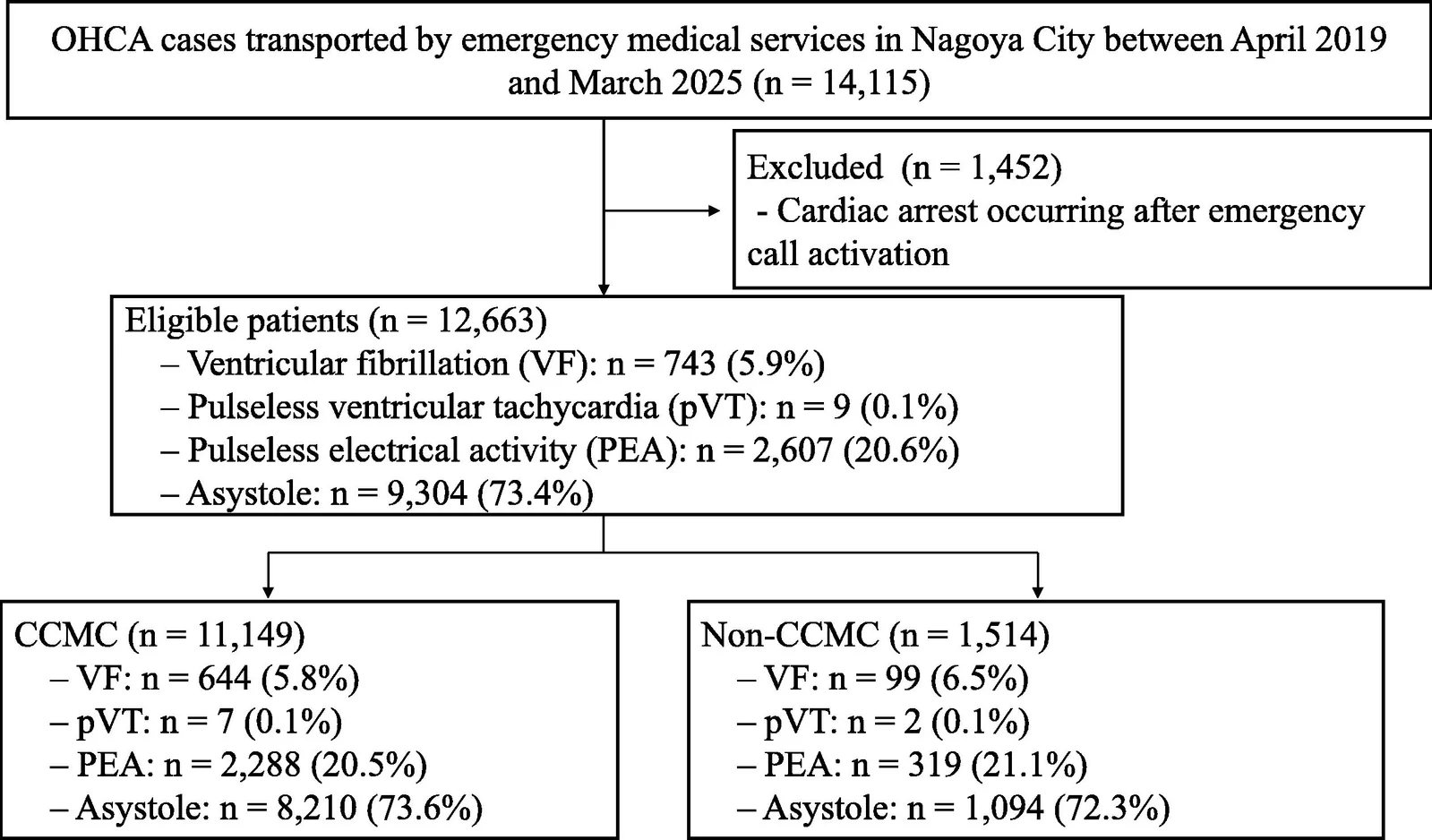
**Study flow diagram of out-of-hospital cardiac arrest (OHCA) patients according to destination hospital level**. CCMC, critical care medical center.

### Data collection

Data were collected from the OHCA record of the Nagoya City Fire Bureau in accordance with the Utstein style.^11^ Collected variables included age, sex, witnessed status, bystander CPR, bystander defibrillation, initial electrocardiographic rhythm, prehospital adrenaline administration, use of a supraglottic airway device, prehospital return of spontaneous circulation (ROSC), destination hospital, one-month survival, and one-month neurological outcome. For EMS-related time variables, we collected the timestamps of the emergency call receipt, dispatch decision, first patient contact by EMS, scene departure, and hospital arrival. For the analysis of refractory shockable rhythm, the number of prehospital defibrillation attempts was also collected.

### Hospital classification and destination decision system

Nagoya City had seven critical care medical centers (CCMCs) throughout the study period, and the number of CCMCs did not change. These centers were generally equipped to provide advanced post-cardiac arrest care, including extracorporeal cardiopulmonary resuscitation (ECPR), PCI, and other forms of comprehensive post-resuscitation care. During the study period, eight additional CCMCs located outside Nagoya City and 21 non-CCMCs also accepted patients with OHCA. Non-CCMCs provide advanced life support according to contemporary resuscitation guidelines, but generally do not offer ECPR. The geographic distribution of these CCMCs and non-CCMC hospitals is shown in Supplementary Fig. 1.

In Nagoya City, a dispatch-guided destination decision system is used for emergency calls suggestive of cardiac arrest, whereby the dispatch center requests acceptance from the nearest hospital available to receive patients with cardiac arrest before the emergency medical team makes contact. The initial request is made regardless of whether the hospital is a CCMC or a non-CCMC and without consideration of patient characteristics or initial electrocardiographic rhythm. No dispatch protocol preferentially directed patients eligible for ECPR or advanced post-cardiac arrest care to a CCMC. Receiving physicians made the final acceptance decision and could decline the request. Patients were transported to the hospital that ultimately accepted them.

### Outcomes

The primary outcome was favorable one-month neurological outcomes, defined as a Cerebral Performance Category (CPC) score of 1 or 2. The secondary outcome was one-month survival.

### Statistical analysis

Patient characteristics and resuscitation variables were compared between the CCMC and non-CCMC groups. Continuous variables are presented as medians with interquartile ranges, and categorical variables as numbers with percentages. Between-group comparisons were performed using the Mann–Whitney *U* test for continuous variables and Fisher’s exact test for categorical variables.

To evaluate the association between destination hospital level and outcomes, inverse probability of treatment weighting (IPTW) was performed as the primary analysis. Propensity score covariates were selected a priori as measurable prehospital confounders. Propensity scores for transport to CCMCs were estimated using logistic regression models including age, sex, witnessed status, bystander CPR, call-to-first patient contact time by EMS, and initial electrocardiographic rhythm in the overall cohort. Separate propensity score models were constructed for shockable and non-shockable rhythms using age, sex, witnessed status, bystander CPR, and call-to-first patient contact time by EMS, with bystander defibrillation included in the shockable model and PEA versus asystole included in the non-shockable model. Transport time was excluded because it may act as both a mediator and collider influenced by destination hospital, prehospital interventions, and unmeasured severity. After weighting, associations between CCMC transport and outcomes were estimated using weighted logistic regression with robust standard errors. Covariate balance before and after weighting was assessed using the standardized mean difference (SMD), with SMD <0.1 considered to indicate adequate balance. Propensity score overlap between groups was also assessed.

As a sensitivity analysis, multivariable logistic regression analyses were performed separately for patients with shockable and non-shockable rhythms. Covariates were selected a priori on the basis of clinical relevance and data availability and included age, sex, witnessed status, bystander CPR, and call-to-first patient contact time by EMS. In analyses restricted to patients with shockable rhythms, bystander defibrillation was additionally included. Because of the limited number of favorable neurological outcomes, PEA and asystole were combined as non-shockable rhythm in these analyses.

As an additional analysis, multivariable logistic regression was performed for patients with prehospital ROSC. An exploratory analysis was also conducted for patients with refractory shockable rhythm, defined as requiring three or more defibrillation attempts before hospital arrival without achieving ROSC. All tests were two-sided, and *P* values <0.05 were considered statistically significant. All statistical analyses were performed using EZR (Saitama Medical Center, Jichi Medical University, Saitama, Japan) and R version 4.5.2.

## Results

### Patient characteristics

During the study period, 14,115 patients with OHCA were transported in Nagoya City. After excluding 1452 patients, 12,663 were included in the final analysis (Fig. 1). Of these, 11,149 patients (88.0%) were transported to CCMCs and 1514 (12.0%) to non-CCMCs. The initial rhythms were ventricular fibrillation (VF) in 743 patients (5.9%), pulseless ventricular tachycardia (pVT) in 9 (0.1%), PEA in 2607 (20.6%), and asystole in 9304 (73.4%).

The patients in the CCMC group were younger than those in the non-CCMC group (CCMC vs. non-CCMC: median age, 80 [interquartile range, 69–87] years vs. 81 [72–88] years, *P* < 0.001). Bystander CPR was more frequent in the non-CCMC group (60.8% vs. 65.3%, *P* < 0.001). The median dispatch decision time was 1 min (interquartile range 1–2) in both groups, without a significant difference (*P* = 0.102). Call-to-first patient contact time by the EMS team and transport time were longer in the CCMC group (*P* < 0.001) (Table 1).

**Table 1:** Baseline characteristics of the study population according to destination hospital level.

	**Overall**			**VF/pVT**			**PEA/asystole**		
	**CCMC (*n* = 11,149)**	**Non-CCMC (*n* = 1514)**	***P* value**	**CCMC (*n* = 651)**	**Non-CCMC (*n* = 101)**	***P* value**	**CCMC (*n* = 10,498)**	**Non-CCMC (*n* = 1413)**	***P* value**
Age	80 [69–87]	81 [72–88]	<0.001	69 [56–77]	69 [58–79]	0.417	80 [70–88]	82 [73–89]	<0.001
Male sex	6236 (55.9%)	883 (58.3%)	0.082	521 (80.0%)	79 (78.2%)	0.690	5715 (54.4%)	804 (56.9%)	0.083
Witnessed cardiac arrest	3122 (28.0%)	411 (27.1%)	0.502	453 (69.6%)	67 (66.3%)	0.563	2669 (25.4%)	344 (24.3%)	0.397
Bystander CPR	6776 (60.8%)	989 (65.3%)	<0.001	423 (65.0%)	66 (65.3%)	1.000	6353 (60.5%)	923 (65.3%)	<0.001
Bystander defibrillation	193 (1.7%)	44 (2.9%)	0.003	52 (8.0%)	11 (10.9%)	0.334	NA	NA	NA
Prehospital adrenaline administration	1263 (11.3%)	153 (10.1%)	0.164	213 (32.7%)	23 (22.8%)	0.050	1050 (10.0%)	130 (9.2%)	0.368
Supraglottic airway use	6572 (58.9%)	854 (56.4%)	0.062	460 (70.7%)	67 (66.3%)	0.414	6112 (58.2%)	787 (55.7%)	0.075
Dispatch decision time	1 [1–2]	1 [1–2]	0.102	1 [1–2]	2 [1–2]	0.534	1 [1–2]	1 [1–2]	0.143
Call-to-first patient contact time by EMS	8 [6–9]	8 [6–10]	<0.001	7 [6–9]	8 [6–9]	0.460	8 [6–9]	8 [7–10]	<0.001
On-scene time	10 [8–12]	10 [8–12]	0.054	11 [8–14]	11 [8–15]	0.577	10 [8–12]	10 [8–12]	0.030
Transport time (scene to hospital)	8 [6–11]	7 [5–9]	<0.001	8 [5–11]	6 [4–9]	<0.001	8 [6–11]	7 [5–9]	<0.001
Call-to-hospital arrival time	25 [21–29]	24 [20–28]	<0.001	25 [21–30]	24 [20–28]	0.075	25 [21–29]	24 [20–28]	<0.001

Values are presented as median [interquartile range] or number (%).

CCMC, critical care medical center; CPR, cardiopulmonary resuscitation; EMS, emergency medical services; NA, not applicable; PEA, pulseless electrical activity; pVT, pulseless ventricular tachycardia; VF, ventricular fibrillation.

### Clinical outcomes

One-month CPC data were available for 12,660 of 12,663 patients (99.98%). In the overall cohort, the favorable one-month neurological outcome (CPC 1–2) rate was 1.9% in the CCMC group and 1.7% in the non-CCMC group, with no significant difference (*P* = 0.683). The one-month survival rate was also similar between the two groups (CCMC vs. non-CCMC: 4.1% vs. 4.5%, *P* = 0.494) (Table 2). In analyses stratified by initial rhythm, patients with asystole transported to non-CCMCs had a higher one-month survival rate than those transported to CCMCs (0.8% vs. 1.7%, *P* = 0.007).

**Table 2:** Clinical outcomes according to destination hospital level.

		**CCMC (*n* = 11,149)**	**Non-CCMC (*n* = 1514)**	***P* value**
Overall	Return of spontaneous circulation (ROSC) status			<0.001
	Prehospital ROSC	808 (7.2%)	107 (7.1%)	
	In-hospital ROSC	1816 (16.3%)	306 (20.2%)	
	No ROSC	8525 (76.5%)	1101 (72.7%)	
	Cerebral Performance Category (CPC) at 1 month			0.535
	CPC 1	138 (1.2%)	19 (1.3%)	
	CPC 2	69 (0.6%)	6 (0.4%)	
	CPC 3	73 (0.7%)	11 (0.7%)	
	CPC 4	181 (1.6%)	32 (2.1%)	
	CPC 5	10,685 (95.9%)	1446 (95.5%)	
	1-month survival	461 (4.1%)	68 (4.5%)	0.494
	Favorable neurological outcome at 1 month (CPC 1–2)	207 (1.9%)	25 (1.7%)	0.683

		**CCMC (*n* = 651)**	**Non-CCMC (*n* = 101)**	

VF/pVT	ROSC status			0.919
	Prehospital ROSC	233 (35.8%)	36 (35.6%)	
	In-hospital ROSC	198 (30.4%)	29 (28.7%)	
	No ROSC	220 (33.8%)	36 (35.6%)	
	CPC at 1 month			0.732
	CPC 1	107 (16.4%)	16 (15.8%)	
	CPC 2	43 (6.6%)	4 (4.0%)	
	CPC 3	39 (6.0%)	4 (4.0%)	
	CPC 4	62 (9.5%)	8 (7.9%)	
	CPC 5	400 (61.4%)	69 (68.3%)	
	1-month survival	251 (38.6%)	32 (31.7%)	0.225
	Favorable neurological outcome at 1 month (CPC 1–2)	150 (23.0%)	20 (19.8%)	0.524

		**CCMC (*n* = 2288)**	**Non-CCMC (*n* = 319)**	

PEA	ROSC status			0.177
	Prehospital ROSC	413 (18.1%)	54 (16.9%)	
	In-hospital ROSC	583 (25.5%)	97 (30.4%)	
	No ROSC	1292 (56.5%)	168 (52.7%)	
	CPC at 1 month			0.596
	CPC 1	26 (1.1%)	2 (0.6%)	
	CPC 2	25 (1.1%)	1 (0.3%)	
	CPC 3	24 (1.0%)	5 (1.6%)	
	CPC 4	67 (2.9%)	9 (2.8%)	
	CPC 5	2144 (93.8%)	302 (94.7%)	
	1-month survival	142 (6.2%)	17 (5.3%)	0.618
	Favorable neurological outcome at 1 month (CPC 1–2)	51 (2.2%)	3 (0.9%)	0.146

		**CCMC (*n* = 8210)**	**Non-CCMC (*n* = 1094)**	

Asystole	ROSC status			0.002
	Prehospital ROSC	162 (2.0%)	17 (1.6%)	
	In-hospital ROSC	1035 (12.6%)	180 (16.5%)	
	No ROSC	7013 (85.4%)	897 (82.0%)	
	CPC at 1 month			0.023
	CPC 1	5 (0.1%)	1 (0.1%)	
	CPC 2	1 (0.0%)	1 (0.1%)	
	CPC 3	10 (0.1%)	2 (0.2%)	
	CPC 4	52 (0.6%)	15 (1.4%)	
	CPC 5	8141 (99.2%)	1075 (98.3%)	
	1-month survival	68 (0.8%)	19 (1.7%)	0.007
	Favorable neurological outcome at 1 month (CPC 1–2)	6 (0.1%)	2 (0.2%)	0.241

Values are presented as number (%).

CCMC, critical care medical center; PEA, pulseless electrical activity; pVT, pulseless ventricular tachycardia; VF, ventricular fibrillation.

Data on CPC were missing in 3 patients (0.02%).

### Inverse probability of treatment weighting analysis

Covariate balance was adequate after weighting in all three analyses, with all SMDs ≤0.020, and propensity score distributions substantially overlapped between groups (Supplementary Table 1 and Supplementary Fig. 2).

In the overall cohort, transport to CCMCs was not associated with a favorable one-month neurological outcome (OR, 1.16; 95% CI, 0.76–1.76; *P* = 0.501) or one-month survival (OR, 0.86; 95% CI, 0.66–1.13; *P* = 0.280). Among patients with shockable rhythm, transport to CCMCs was not associated with a favorable one-month neurological outcome (OR, 1.13; 95% CI, 0.67–1.91; *P* = 0.649) or one-month survival (OR, 1.30; 95% CI, 0.83–2.04; *P* = 0.254). Among patients with non-shockable rhythm, transport to CCMCs was not associated with a favorable one-month neurological outcome (OR, 1.46; 95% CI, 0.58–3.67; *P* = 0.423), but was associated with lower one-month survival (OR, 0.66; 95% CI, 0.46–0.95; *P* = 0.026) (Table 3).

**Table 3:** Association between destination hospital level and outcomes using inverse probability of treatment weighting.

	**Outcome**	**OR for CCMC transport**	**95% CI**	***P* value**
Overall cohort	Favorable neurological outcome (CPC 1–2)	1.16	0.76–1.76	0.501
	1-month survival	0.86	0.66–1.13	0.280

Shockable rhythm	Favorable neurological outcome (CPC 1–2)	1.13	0.67–1.91	0.649
	1-month survival	1.30	0.83–2.04	0.254

Non-shockable rhythm	Favorable neurological outcome (CPC 1–2)	1.46	0.58–3.67	0.423
	1-month survival	0.66	0.46–0.95	0.026

CCMC, critical care medical center; CI, confidence interval; CPC, Cerebral Performance Category; OR, odds ratio.

### Sensitivity analysis using multivariable logistic regression

Among patients with shockable rhythms (VF/pVT), transport to CCMCs was not significantly associated with favorable one-month neurological outcomes (adjusted odds ratio [aOR], 1.19; 95% confidence interval [CI], 0.68–2.07; *P* = 0.542) or one-month survival (aOR, 1.32; 95% CI, 0.82–2.12; *P* = 0.248). Among patients with PEA/asystole, transport to CCMCs was not significantly associated with a favorable neurological outcome (aOR, 1.41; 95% CI, 0.56–3.53; *P* = 0.467). However, transport to CCMCs was associated with lower one-month survival (aOR, 0.68; 95% CI, 0.47–0.98; *P* = 0.039) (Table 4). Witnessed arrest was consistently associated with better outcomes across rhythm categories, whereas bystander CPR was associated with favorable neurological outcomes and one-month survival only among patients with shockable rhythms.

**Table 4:** Sensitivity analysis using multivariable logistic regression according to initial rhythm.

	**VF/pVT**			**PEA/Asystole**		
**Variable**	**aOR**	**95% CI**	***P* value**	**aOR**	**95% CI**	***P* value**
**A. 1-month favorable neurological outcome (CPC 1–2)**						
CCMC vs Non-CCMC	1.19	0.68–2.07	0.542	1.41	0.56–3.53	0.467
Age	0.97	0.95–0.98	<0.001	0.99	0.97–0.997	0.016
Male sex	1.39	0.85–2.28	0.194	1.72	0.99–2.98	0.054
Witnessed cardiac arrest	2.00	1.29–3.11	0.002	4.39	2.64–7.32	<0.001
Bystander CPR	2.44	1.59–3.76	<0.001	1.01	0.60–1.69	0.973
Bystander defibrillation	1.09	0.59–2.02	0.788	NA	NA	NA
Call-to-first patient contact time by EMS	0.90	0.83–0.98	0.010	0.89	0.81–0.99	0.034

**B. 1-month survival**						
CCMC vs Non-CCMC	1.32	0.82–2.12	0.248	0.68	0.47–0.98	0.039
Age	0.97	0.96–0.98	<0.001	0.98	0.97–0.98	<0.001
Male sex	1.17	0.78–1.76	0.436	1.16	0.89–1.51	0.280
Witnessed cardiac arrest	1.92	1.34–2.74	<0.001	4.41	3.40–5.71	<0.001
Bystander CPR	1.78	1.26–2.52	0.001	1.04	0.80–1.36	0.769
Bystander defibrillation	1.07	0.61–1.89	0.806	NA	NA	NA
Call-to-first patient contact time by EMS	0.91	0.85–0.97	0.004	0.92	0.87–0.96	<0.001

Values are adjusted odds ratios (aORs) with 95% confidence intervals (CIs).

CCMC, critical care medical center; CPC, Cerebral Performance Category; CPR, cardiopulmonary resuscitation; EMS, emergency medical services; NA, not applicable; PEA, pulseless electrical activity; pVT, pulseless ventricular tachycardia; VF, ventricular fibrillation.

### Additional analysis in patients with prehospital return of spontaneous circulation

In an additional multivariable analysis restricted to patients with prehospital ROSC, transport to CCMCs was not associated with favorable one-month neurological outcomes (aOR, 1.07; 95% CI, 0.60–1.91; *P* = 0.815) or one-month survival (aOR, 0.74; 95% CI, 0.45–1.20; *P* = 0.220) (Supplementary Table 2).

### Exploratory analysis of refractory shockable rhythm

We analyzed 253 patients with refractory shockable rhythm who required three or more defibrillation attempts before hospital arrival without achieving ROSC. The one-month survival rate was higher in the CCMC group than in the non-CCMC group (19.7% vs. 3.3%; OR, 7.10; 95% CI, 1.11–297; *P* = 0.023). The favorable one-month neurological outcome rate was also higher in the CCMC group than in the non-CCMC group (6.7% vs. 0%); however, the difference was not statistically significant (Table 5).

**Table 5:** Outcomes in patients with refractory shockable rhythm.

	**CCMC (*n* = 223)**	**Non-CCMC (*n* = 30)**	**Crude odds ratio (95% CI)**	***P* value**
1-month survival	44 (19.7%)	1 (3.3%)	7.10 (1.11–297)	0.023
Favorable neurological outcome (CPC 1–2)	15 (6.7%)	0 (0%)	∞ (0.49–∞)	0.228

Fisher’s exact test was used for comparisons.

Refractory shockable rhythm was defined as ≥3 prehospital defibrillation attempts without return of spontaneous circulation prior to hospital arrival.

Exact confidence intervals (CIs) are shown for favorable neurological outcome because no events occurred in the non-CCMC group.

CCMC, critical care medical center; CPC, Cerebral Performance Category.

## Discussion

This was a population-based observational study conducted in Nagoya City, where a dispatch-guided transport system is used, in which the initial acceptance request is made to the nearest hospital available to receive patients with cardiac arrest, regardless of the patient’s characteristics or initial electrocardiographic rhythm. We examined the association between destination hospital level and patient outcomes among all patients with OHCA transported during the study period. Transport to CCMCs was not significantly associated with favorable one-month neurological outcomes in the overall cohort, but its association with one-month survival appeared to differ according to the initial rhythm. In particular, transport to CCMCs was associated with lower one-month survival among patients with non-shockable rhythm, although it was not associated with favorable neurological outcomes. In an exploratory analysis of patients with refractory shockable rhythm, one-month survival was higher in the CCMC group, although the estimate was imprecise.

Regarding the association between destination hospital level and outcomes after OHCA, previous studies have suggested that transport to cardiac arrest centers (CACs) may be associated with improved outcomes.^6^, ^9^, ^12^, ^13^, ^14^ A Japanese registry-based study likewise showed that transport to CCMCs was independently associated with favorable neurological outcome.^15^ One possible explanation for these associations is that advanced treatments, such as coronary angiography, PCI, targeted temperature management, and mechanical circulatory support, are more frequently performed at CACs.^14^, ^16^, ^17^, ^18^, ^19^ On the other hand, recent studies have suggested that some of these differences may be explained by variations in patient characteristics and prehospital factors.^20^, ^21^, ^22^ In other words, OHCA outcomes are strongly determined by prehospital factors such as initial rhythm, witnessed status, bystander CPR, and resuscitation duration, and the hospital level itself may not be a consistent independent determinant of these outcomes.^8^, ^20^, ^21^, ^22^, ^23^, ^24^ In the present study as well, witnessed status and bystander CPR, rather than destination hospital level, were shown to influence neurological outcomes and survival, which is consistent with previous reports. Confounding by indication has also been recognized as a potential source of bias in previous observational studies evaluating cardiac arrest center transport.^25^ In Nagoya City, the initial hospital request is made before EMS contact without consideration of patient characteristics or initial electrocardiographic rhythm, which may limit the opportunity for such confounding at the initial request. However, confounding by indication during the subsequent hospital acceptance process could not be assessed or excluded because information on the hospitals requested, the number and sequence of requests, and hospital refusals was unavailable.

Recent evidence suggests that any benefit of transport to CACs may be limited to selected patient groups with a relatively higher likelihood of a favorable outcome.^24^ In the exploratory analysis of patients with refractory shockable rhythm, the one-month survival rate was higher in the CCMC group. One possible explanation is the availability of advanced circulatory support therapies at CCMCs, including ECPR. In patients with shockable rhythm, reversible conditions such as acute coronary syndrome are common, and advanced therapies such as PCI and intensive care may contribute to improved outcomes. Recent guidelines suggest that, in addition to high-quality resuscitation at the scene, early transport to specialized centers may be beneficial for patients after ROSC or for those in whom ECPR is being considered.^26^ An observational study from Kobe, Japan, reported that among patients with OHCA due to refractory VF/VT, transport to ECPR-capable hospitals was associated with improved favorable neurological outcomes and survival.^27^ In addition, an international comparative study of patients with OHCA and shockable rhythm showed regional differences in neurological outcomes and suggested that variations in in-hospital treatment systems, including the availability of ECPR, may have contributed to these differences.^28^ Our findings raise the hypothesis that comprehensive treatment, including advanced circulatory support, may be associated with improved survival in patients with refractory shockable rhythm, although the estimate was imprecise because of the small non-CCMC group.

However, in an additional analysis of patients with prehospital ROSC, transport to CCMCs was not associated with improved neurological or survival outcomes. Likewise, among patients with non-shockable rhythm, transport to CCMCs was not significantly associated with favorable neurological outcomes. Furthermore, transport to CCMCs was associated with lower one-month survival in patients with non-shockable rhythm. In non-shockable rhythm, prolonged low-flow time and non-cardiac etiologies are common, and the potential for treatment to improve outcomes has been considered limited.^8^ In fact, a nationwide registry study of patients with asystole reported that the 30-day favorable neurological outcome rate was extremely low at 0.2%, and that although prehospital adrenaline administration and airway management were associated with survival, they were not associated with favorable neurological outcomes.^29^ Unmeasured differences in hospital acceptance or in-hospital resuscitation practices may have affected this finding.

In this dispatch-guided transport system, no clear overall association between CCMC transport and better outcomes was observed. However, this study did not directly compare alternative transport strategies and therefore cannot determine which transport strategy is preferable. One possible explanation for these findings is that resuscitation management and post-resuscitation care based on recent resuscitation guidelines may have become increasingly standardized even in non-CCMC hospitals.^5^, ^26^, ^30^, ^31^ On the other hand, transport to a CCMC may be associated with better outcomes in patients with refractory shockable rhythm; however, because this finding was based on an exploratory analysis with a small number of patients, further studies are needed to determine whether such patients should be preferentially transported to CCMCs.

A major strength of this study is that it was a population-based study including all patients with OHCA in Nagoya City. We evaluated a complete citywide cohort of EMS-transported OHCA cases during the study period, which addressed limitations of previous hospital-based studies. Another strength was near-complete one-month CPC ascertainment (12,660/12,663; 99.98%). Another strength is that we used dispatch-guided system data. Under the dispatch-guided system, the initial acceptance request is made to the nearest hospital available to receive patients with cardiac arrest before EMS contact, without consideration of patient characteristics or initial electrocardiographic rhythm.

This study had several limitations. First, because this was a retrospective observational study, unmeasured confounding cannot be excluded. Arrest location was unavailable for adjustment. In addition, selection bias at the hospital acceptance stage could not be excluded because data on acceptance requests and refusals were unavailable. The number and sequence of hospital requests were not recorded. Therefore, differences in the hospital acceptance process between the CCMC and non-CCMC groups could not be assessed. Second, data on in-hospital treatments such as PCI, targeted temperature management, and ECPR were unavailable, preventing full evaluation of their impact on outcomes. Third, classification as CCMC or non-CCMC may not fully reflect differences in resources and treatment capabilities. Although CCMC numbers and the dispatch protocol remained unchanged, data on hospital acceptance policies, ECPR capability, and post-cardiac arrest care were unavailable. Fourth, the exploratory analysis of refractory shockable rhythm included only 30 non-CCMC patients and one survivor, yielding a very wide confidence interval and substantial uncertainty. Finally, because this study was based on data from a single metropolitan area, the findings should be generalized to other regions with caution.

## Conclusion

In patients with OHCA, CCMC transport was not clearly associated with favorable neurological outcomes in either shockable or non-shockable rhythms. However, exploratory findings suggested a potential association between CCMC transport and better outcomes in patients with refractory shockable rhythm, although the small sample size and imprecise estimates warrant further investigation.

## Sources of funding

None.

## CRediT authorship contribution statement

**Akihiro Kato:** Writing – review & editing, Writing – original draft, Validation, Methodology, Investigation, Formal analysis, Conceptualization. **Asako Matsushima:** Writing – review & editing, Validation, Supervision, Project administration. **Momoko Sugiyama:** Writing – review & editing, Validation, Data curation. **Akito Kubota:** Writing – review & editing, Validation, Data curation. **Kazunori Imai:** Writing – review & editing, Validation, Data curation. **Toshiyasu Miura:** Writing – review & editing, Validation, Data curation. **Yasumasa Ode:** Writing – review & editing, Validation, Data curation.

## Declaration of competing interest

The authors declare no conflicts of interest in association with the present study.
