# Supplementary material for: Association between destination hospital level and outcomes in out-of-hospital cardiac arrest patients under a dispatch-guided transport system: a population-based study in Nagoya, Japan
Source: Resusc Plus. 2026 Sep 8;32:101482. doi: 10.1016/j.resplu.2026.101482 (PMC13625888; doi:10.1016/j.resplu.2026.101482)

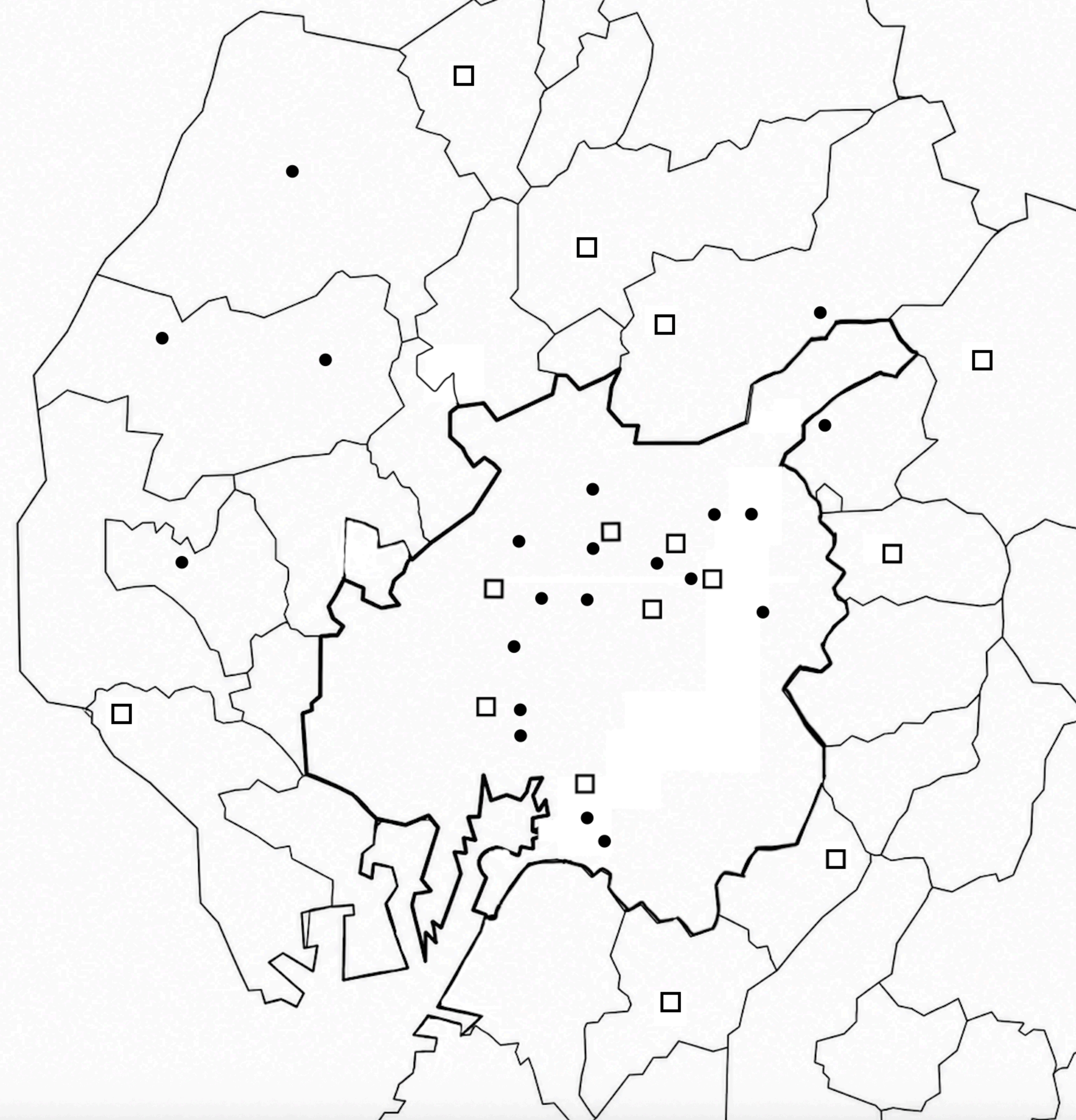

Supplement: Supplementary file 1 [file mmc1.pdf]
