# Supplementary material for: Association between destination hospital level and outcomes in out-of-hospital cardiac arrest patients under a dispatch-guided transport system: a population-based study in Nagoya, Japan
Source: Resusc Plus. 2026 Sep 8;32:101482. doi: 10.1016/j.resplu.2026.101482 (PMC13625888; doi:10.1016/j.resplu.2026.101482)
Supplement: Supplementary file 2 [file mmc2.pdf]

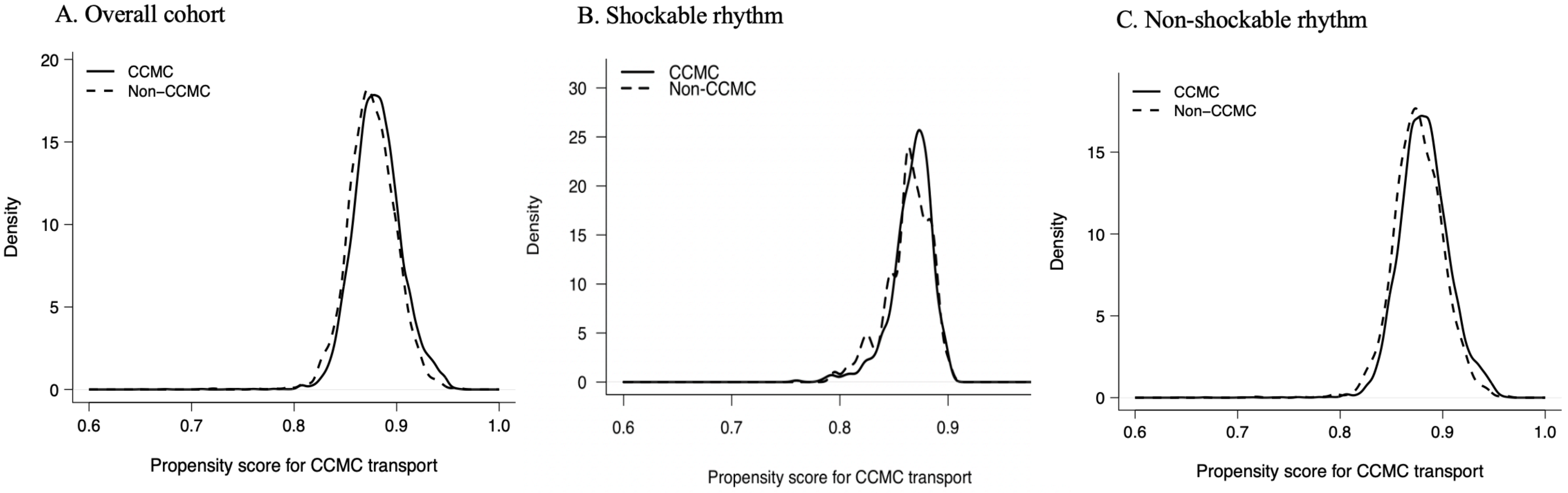
A. Overall cohort
CCMC
Non–CCMC
Density
Propensity score for CCMC transport
B. Shockable rhythm
CCMC
Non-CCMC
Density
Propensity score for CCMC transport
C. Non-shockable rhythm
CCMC
Non–CCMC
Density
Propensity score for CCMC transport
