# Supplementary material for: Association between destination hospital level and outcomes in out-of-hospital cardiac arrest patients under a dispatch-guided transport system: a population-based study in Nagoya, Japan
Source: Resusc Plus. 2026 Sep 8;32:101482. doi: 10.1016/j.resplu.2026.101482 (PMC13625888; doi:10.1016/j.resplu.2026.101482)
Supplement: Supplementary file 3 [file mmc3.docx]

Supplementary Table 1. Covariate balance before and after inverse probability of treatment weighting (IPTW)

| A. Overall cohort |  |  |
| --- | --- | --- |
| Variable | SMD before IPTW | SMD after IPTW |
| Age | 0.157 | 0.004 |
| Male sex | 0.048 | 0.001 |
| Witnessed cardiac arrest | 0.019 | 0.003 |
| Bystander CPR | 0.094 | 0.003 |
| Call-to-first patient contact time by EMS | 0.094 | 0.006 |
| Shockable rhythm | 0.034 | 0.005 |
|  |  |  |
| B. Shockable rhythm |  |  |
| Variable | SMD before IPTW | SMD after IPTW |
| Age | 0.082 | 0.016 |
| Male sex | 0.044 | 0.012 |
| Witnessed cardiac arrest | 0.069 | 0.001 |
| Bystander CPR | 0.008 | 0.018 |
| Bystander defibrillation | 0.099 | 0.000 |
| Call-to-first patient contact time by EMS | 0.070 | 0.001 |
|  |  |  |
| C. Non-shockable rhythm |  |  |
| Variable | SMD before IPTW | SMD after IPTW |
| Age | 0.169 | 0.005 |
| Male sex | 0.050 | 0.001 |
| Witnessed cardiac arrest | 0.025 | 0.003 |
| Bystander CPR | 0.100 | 0.006 |
| Call-to-first patient contact time by EMS | 0.098 | 0.006 |
| PEA versus asystole | 0.019 | 0.006 |

CPR, cardiopulmonary resuscitation; EMS, emergency medical services; IPTW, inverse probability of treatment weighting; PEA, pulseless electrical activity; SMD, standardized mean difference.

Supplementary Table 2. Multivariable logistic regression analyses for 1-month favorable neurological outcome and 1-month survival among patients with prehospital return of spontaneous circulation (ROSC)

| A. Favorable neurological outcome (CPC 1–2) |  |  |  |
| --- | --- | --- | --- |
|  | Overall | | |
| Variable | aOR | 95% CI | P value |
| CCMC vs Non-CCMC | 1.07 | 0.60–1.91 | 0.815 |
| Shockable rhythm | 8.64 | 5.88–12.70 | <0.001 |
| Age | 0.97 | 0.95–0.98 | <0.001 |
| Male sex | 1.60 | 1.03–2.48 | 0.038 |
| Witnessed cardiac arrest | 1.17 | 0.77–1.80 | 0.463 |
| Bystander CPR | 1.06 | 0.70–1.62 | 0.773 |
| Call-to-first patient contact time by EMS | 0.97 | 0.90–1.06 | 0.503 |
|  |  |  |  |
|  |  |  |  |
| B. 1-month survival |  |  |  |
|  | Overall | | |
| Variable | aOR | 95% CI | P value |
| CCMC vs Non-CCMC | 0.74 | 0.45–1.20 | 0.220 |
| Shockable rhythm | 7.07 | 4.97–10.10 | <0.001 |
| Age | 0.97 | 0.96–0.98 | <0.001 |
| Male sex | 1.09 | 0.77–1.53 | 0.629 |
| Witnessed cardiac arrest | 1.50 | 1.05–2.15 | 0.026 |
| Bystander CPR | 0.87 | 0.62–1.24 | 0.447 |
| Call-to-first patient contact time by EMS | 0.94 | 0.88–1.00 | 0.053 |

Values are adjusted odds ratios (aORs) with 95% confidence intervals (CIs).

CCMC, critical care medical center; CPC, Cerebral Performance Category; CPR, cardiopulmonary resuscitation; EMS, emergency medical services.
